# Landscape of T-cell engagers in solid tumors

**DOI:** 10.1093/oncolo/oyag129

**Published:** 2026-04-08

**Authors:** Esther Garcia-Lorenzo, Miriam Dorta, Bernard Doger, Manuel Pedregal, Victor Moreno

**Affiliations:** START Madrid-FJD, Early Phase Clinical Trials Unit, Hospital Universitario Fundación Jiménez Díaz, Madrid, Spain; Instituto Investigación Sanitaria Hospital Fundación Jiménez Díaz (IIS-FJD), Madrid, Spain; START Madrid-FJD, Early Phase Clinical Trials Unit, Hospital Universitario Fundación Jiménez Díaz, Madrid, Spain; Instituto Investigación Sanitaria Hospital Fundación Jiménez Díaz (IIS-FJD), Madrid, Spain; START Madrid-FJD, Early Phase Clinical Trials Unit, Hospital Universitario Fundación Jiménez Díaz, Madrid, Spain; Instituto Investigación Sanitaria Hospital Fundación Jiménez Díaz (IIS-FJD), Madrid, Spain; START Madrid-FJD, Early Phase Clinical Trials Unit, Hospital Universitario Fundación Jiménez Díaz, Madrid, Spain; Instituto Investigación Sanitaria Hospital Fundación Jiménez Díaz (IIS-FJD), Madrid, Spain; START Madrid-FJD, Early Phase Clinical Trials Unit, Hospital Universitario Fundación Jiménez Díaz, Madrid, Spain; Instituto Investigación Sanitaria Hospital Fundación Jiménez Díaz (IIS-FJD), Madrid, Spain

**Keywords:** T-cell engagers, bispecific antibodies, immunotherapy, solid tumors, cytokine release syndrome

## Abstract

T-cell engagers (TCEs) are a diverse class of bispecific and multispecific molecules that co-bind CD3 on T cells and tumor-associated antigens to form an immune synapse and induce targeted T-cell–mediated cytotoxicity. While TCEs have demonstrated remarkable efficacy in hematologic malignancies, translation into solid tumors has been more challenging. Recent advances seen with tebentafusp in metastatic uveal melanoma and tarlatamab in small-cell lung cancer have validated the approach and driven a rapidly expanding pipeline targeting other tumor associated antigens such as STEAP1, MUC16, and PRAME among others. Unique challenges in solid tumors include antigen heterogeneity and density thresholds, on-target/off-tumor toxicities, and physical and immunologic barriers within the tumor microenvironment. To address these, next-generation engineering strategies, such as half-life extension, protease- or context-dependent masking, multispecificity, and “armed” constructs incorporating cytokine or co-stimulatory payloads, are being developed to enhance intratumoral activity while limiting systemic toxicities. Combination regimens with checkpoint blockade, chemotherapy, targeted therapies, and oncolytic platforms are also being actively investigated to overcome immune resistance and improve durability of response. Collectively, next-generation TCEs guided by rational target selection, context-dependent activation, and biomarker-driven patient stratification, are poised to broaden the reach of immunotherapy in solid tumors. In this review, we synthesize the recent advances that aim to expand the therapeutic window of TCEs for the treatment of solid tumors.

Implications for practice T-cell engagers are an innovative class of immunotherapies that redirect T lymphocytes toward cancer cells. Although highly effective in hematologic malignancies, their success in solid tumors has been more limited. Tebentafusp and tarlatamab have provided clinical proof of concept in uveal melanoma and small cell lung cancer, respectively. Early-phase trials have also shown antitumor activity with other T-cell engagers in prostate, ovarian, and gastrointestinal cancers among others. Further progress will depend on improved target selection, minimize risk of severe toxicities like cytokine release syndrome, and rational combinations that enhance efficacy without compromising safety.

## Introduction

Immunotherapy has brought a paradigm shift in cancer treatment, fundamentally transforming therapeutic strategies and reshaping our understanding of tumor–immune system interactions. The clinical success of immune checkpoint inhibitors (ICIs), particularly those targeting CTLA-4, PD-1, and more recently LAG-3, has revolutionized the management of several malignancies, offering durable responses in patients with historically poor prognoses.^1–3^

In parallel, T cell–based immunotherapies such as tumor-infiltrating lymphocytes (TILs), engineered T cell receptors (TCRs), and bispecific molecules like tebentafusp, a gp100xCD3 *Immune-mobilizing monoclonal TCRs Against Cancer* (ImmTAC), have expanded the therapeutic landscape, particularly in melanoma and other solid tumors.^4–6^ These modalities harness the cytotoxic potential of T cells and have demonstrated encouraging efficacy in selected indications.

However, long-term benefit remains limited to a fraction of patients. Resistance to ICIs (both primary and acquired) poses a major clinical challenge, often driven by tumor-intrinsic mechanisms such as low tumor mutational burden, impaired antigen presentation via MHC downregulation, and immune escape through clonal selection.^7^^,^^8^ These limitations highlight the need for alternative strategies that can bypass conventional antigen presentation pathways and effectively redirect T cell activity.

One of the key insights from recent advances in immunotherapy is the pivotal role of T cells in achieving durable tumor control, even in metastatic settings, as demonstrated in long-term survivors treated with ICIs in melanoma and lung cancer.^1^^,^^2^ This recognition has driven the development of T cell engagers (TCEs), a novel class of bispecific molecules designed to simultaneously bind CD3 on T cells and a tumor-associated surface antigen (TAA), thereby triggering targeted cytotoxicity. Unlike ICIs, TCEs do not require recognition of peptide–MHC complexes, positioning them as promising tools to overcome established mechanisms of immune resistance. By enabling T cell activation and tumor killing in an antigen-specific but MHC-independent manner, TCEs may offer new therapeutic opportunities for malignancies with poor immunogenicity or impaired antigen presentation, settings where current immunotherapies often fail.^9^

In this review, we provide a focused overview of T cell engagers in solid tumors, outlining their structure and mechanism of action, preclinical and clinical development, and the specific challenges associated with their use in solid tumor contexts.

## Brief history of T cell engagers

The concept of redirecting T cells to eliminate tumor cells using bispecific antibodies originated in the 1990s. However, early constructs suffered from pharmacokinetic limitations, limited stability, and significant on-target/off-tumor toxicity.^10^^,^^11^ The breakthrough came with the development and subsequent approval of blinatumomab (Blincyto), a CD19-CD3 bispecific T cell engager (BiTE), for relapsed/refractory B-cell acute lymphoblastic leukemia (B-ALL). Blinatumomab received accelerated approval by the FDA in 2014 and full approval in 2017 after demonstrating superior overall survival compared to chemotherapy in a phase 3 trial.^12^

This milestone validated the bispecific TCE platform and stimulated a wave of innovation. Subsequent approvals of bispecific agents in hematologic malignancies, such as teclistamab and elranatamab (both targeting BCMA in multiple myeloma), as well as glofitamab and epcoritamab (targeting CD20 in lymphoma), have further consolidated the clinical relevance of this strategy.^13–16^ Importantly, B cells represent a unique therapeutic target because their near-complete eradication is clinically manageable, given the possibility of immunoglobulin replacement. This feature distinguishes them from most other cellular targets, where antigen loss or depletion would result in severe, often unacceptable toxicities, and helps explain why the first clinical breakthroughs with TCEs occurred in B-cell malignancies.

More recently, the approval of tarlatamab, a DLL3xCD3 bispecific TCE for the treatment of small cell lung cancer (SCLC) marked a turning point for the application of these therapies in solid tumors.^17^ Advances in molecular engineering have enabled the development of half-life extended molecules (ie, tarlatamab), dual-targeting constructs, and conditionally activated TCEs designed to improve pharmacokinetics, safety, and tumor specificity in solid oncology.^9^^,^^18^

## Structure and mechanism of action of T-cell engagers

TCEs are engineered antibody-based molecules as their name implies, redirect T cells toward tumor cells. They typically achieve this by simultaneously binding CD3 on T cells and a tumor-associated antigen (TAA) on malignant cells, thus forming a cytolytic immunological synapse that activates T cells. This process occurs independently of classical peptide-MHC presentation and co-stimulatory signaling.^9^^,^^19^

**Figure 1:**
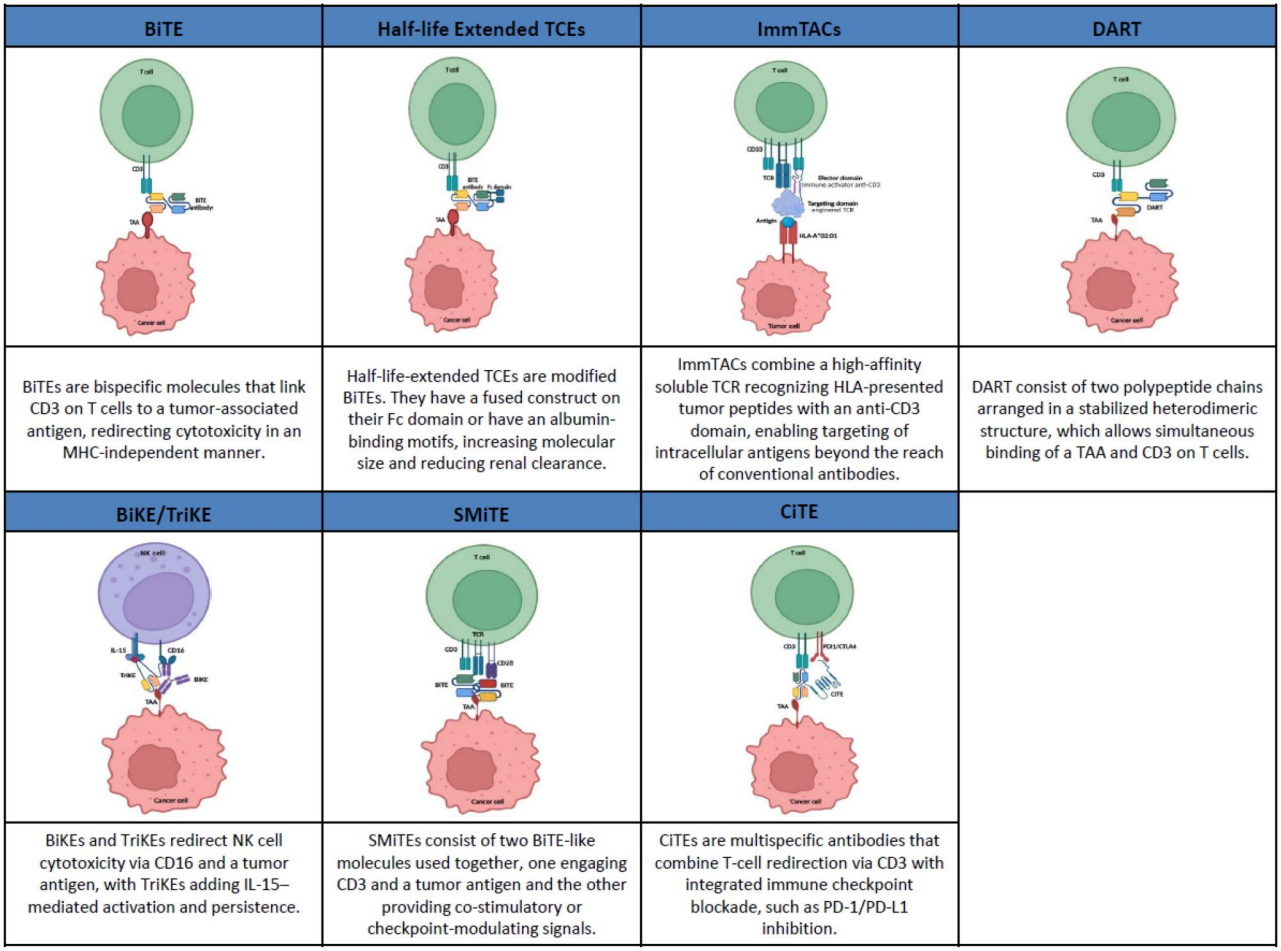
Differences in structure and mechanism of action of the different T-cell engagers.

However, a distinct subset of T-cell–redirecting therapies, such as TCR-based engagers and ImmTACs, relies on recognition of intracellular tumor-derived peptides presented by specific HLA molecules (Figure 1).^20^^,^^21^

### BiTEs (bispecific T-cell engagers)

The first clinically successful TCEs were BiTEs, such as blinatumomab, composed of 2 single-chain variable fragments (scFvs): one targeting CD3 and the other a tumor antigen.^20^ This small, flexible structure allows efficient immunological synapse formation and potent cytotoxicity. Once CD3 is engaged, T cells are activated to release perforin and granzymes, leading to tumor cell lysis. Importantly, this mechanism bypasses the need for peptide–MHC recognition, allowing T cell redirection even in tumors with defective antigen presentation or low MHC expression.^9^^,^^10^^,^^19^

### Half-life extended TCEs

To overcome the short half-life and infusion-related limitations of canonical BiTEs, half-life extended TCEs have been developed. These constructs typically fuse the bispecific antibody fragment to an Fc domain or use alternative scaffold technologies to prolong systemic exposure and enable intermittent or outpatient dosing.^18^ A notable example is tarlatamab, a DLL3xCD3 bispecific TCE with a modified Fc region, which demonstrated meaningful activity and manageable safety in small cell lung cancer (SCLC).^17^

### ImmTACs (immune mobilizing monoclonal TCRs against cancer) and other MHC-dependent T-cell engagers

ImmTACs represent a mechanistically distinct class of T-cell–redirecting therapies that rely on the recognition of intracellular tumor-derived peptides presented by specific HLA molecules, rather than direct binding to cell surface TAAs. Conceptually, these agents are more closely related to engineered TCR-based therapies than to classical MHC-independent CD3×tumor-associated antigen (TAA) bispecific antibodies, analogous to the distinction between TCR-T cells and CAR-T cells.^20^^,^^21^

These molecules combine a soluble, high-affinity engineered TCR specific for a defined peptide–HLA complex with an anti-CD3 single-chain variable fragment, thereby redirecting polyclonal T cells toward tumor cells expressing intracellular antigens. This strategy expands the range of targetable antigens beyond cell surface proteins to include differentiation antigens and cancer-testis antigens, such as gp100 and PRAME, and in principle could be extended to recurrent oncogenic neoantigens, including mutant KRAS–derived peptides.^20^^,^^22^

However, the activity of ImmTACs and other MHC-dependent T-cell engagers is strictly contingent on intact HLA expression and a functional antigen processing and presentation machinery. As a result, immune escape mechanisms such as HLA loss, β2-microglobulin downregulation, or defects in peptide loading represent key vulnerabilities of this therapeutic class. In addition, canonical TCR signaling induced by these agents is associated with rapid upregulation of inhibitory immune checkpoints, including PD-1, providing a strong mechanistic rationale for combination strategies with PD-(L)1 blockade.^22^^,^^23^

Clinical experience with tebentafusp, an ImmTAC targeting gp100 presented by HLA-A*02:01, has demonstrated a significant overall survival benefit in metastatic uveal melanoma despite low RECIST-defined objective response rates. These findings suggest that ImmTAC-mediated antitumor activity may extend beyond direct tumor cytotoxicity and involve broader immunomodulatory and immune-priming effects within the tumor microenvironment, and that durable clinical benefit can be achieved in selected settings without mandatory concomitant checkpoint inhibition.^6^^,^^24^

Finally, preclinical evidence indicates that, unlike some checkpoint-based immunotherapies, T-cell engagers—including MHC-dependent platforms such as ImmTACs—do not consistently induce antigen spreading. Consequently, therapeutic efficacy remains closely linked to sustained expression of the targeted peptide–HLA complex, potentially limiting durability of response and predisposing tumors to relapse through antigen loss or clonal selection.^8^

### Other advanced formats

Beyond BiTEs and ImmTACs, other TCE platforms include:

- **DARTs** (Dual-Affinity Re-Targeting molecules): stabilized diabody-like structures designed to enhance pharmacokinetics and molecular stability.^25^
- **BiKEs/TriKEs**: recruit NK cells instead of T cells by targeting CD16, with TriKEs often including an IL-15 moiety to boost NK cell activity and persistence.^26^
- **SMITEs**: combine 2 BiTE-like molecules, one delivering TCR activation via CD3 and the other co-stimulation through CD28.^27^
- **CiTEs**: merge checkpoint blockade and T cell redirection in a single molecule, potentially enhancing efficacy in immunosuppressive microenvironments.^28^

These next-generation platforms aim to improve tumor specificity, expand the therapeutic index, and mitigate on-target off-tumor toxicities, which are particularly relevant challenges in solid tumor settings.

## Preclinical development and optimization

Although T cell engager therapies hold substantial promise, especially for solid tumors, their development faces critical preclinical challenges. Chief among these is the identification of TAAs that are highly expressed in tumors, minimally present in normal tissues, and capable of mediating durable antitumor responses while avoiding clinically relevant off-tumor toxicity. However, antigen heterogeneity, antigen loss, and an immunosuppressive tumor microenvironment often hinder efficacy and contribute to resistance.^8^^,^^29^

The scarcity of approved TAA-targeted immunotherapies in solid tumors reflects the biological complexity of target selection. Antigen expression in normal tissues carries a non-negligible risk of severe immune-related adverse events, including on-target, off-tumor toxicities that may limit dose escalation or lead to early treatment discontinuation^30^

From a structural perspective, canonical BiTE molecules are compact molecules that spans approximately 65 Å, enabling it to bridge T cells and tumor cells within the physical space of an immunological synapse, typically around 130 Å.^31^ This spatial efficiency drives high-potency cytotoxic activity at low concentrations (10-100 pg/mL).^19^

However, the short serum half-life of canonical BiTEs is primarily driven by the absence of an Fc domain and the resulting lack of FcRn-mediated recycling. While this small size can be advantageous for synapse formation and tumor penetration, it necessitated continuous infusion for first-generation agents like blinatumomab. To overcome this limitation, newer constructs incorporate half-life extension strategies, including albumin-binding domains or Fc-fusions.^9^ Additional stabilization techniques, including disulfide engineering, directed evolution, and VH-VL interface stabilization, have been applied to enhance molecular durability and manufacturing robustness.^9^

Further complication arises from the mechanism of action itself. Engagement of CD3 and subsequent T cell activation often leads to the release of pro-inflammatory cytokines, which can induce cytokine release syndrome (CRS), a potentially life-threatening adverse event.^32^ Preclinical models have limited ability to predict the incidence and severity of CRS accurately, making translational safety assessment a key bottleneck in TCE development.

Overall, preclinical optimization, spanning target validation, construct engineering, stability enhancement, and immune toxicity modeling, remains essential for the successful clinical translation of T-cell engagers.

## Clinical data: from hematologic to solid tumors

TCEs have gained considerable attention as a new class of immunotherapeutics capable of redirecting cytotoxic lymphocytes toward tumor cells. Most of the clinical development to date has focused on hematologic malignancies, where TCEs have shown favorable pharmacodynamics, consistent antigen expression, and minimal tumor architecture barriers.

The first TCE approved by the FDA, blinatumomab, demonstrated a complete remission rate of 43-44% and a median overall survival benefit over chemotherapy in randomized trials.^12^^,^^31^ Since then, several other TCEs have been developed and approved for hematologic indications, including teclistamab, elranatamab, and talquetamab (targeting BCMA and GPRC5D in multiple myeloma), as well as glofitamab and epcoritamab (targeting CD20 in B-cell lymphomas).^13^^,^^14^^,^^16^^,^^33^ Nevertheless, most TCE candidates remain in early-phase trials (phase 1-2 trials) where safety and dose optimization are the primary endpoints.

### DLL3 in small cell lung cancer (SCLC)

Of the solid tumor trials conducted to date, the DLL3-targeted TCE tarlatamab (AMG 757) has garnered particular attention for its promising activity and safety profile in SCLC. DLL3 is aberrantly expressed on SCLC cells and has minimal expression in normal tissues, making it an attractive target.

In the phase 1 study (NCT03319940), tarlatamab showed an objective response rate (ORR) of 23.4% **(**95% CI, 15.7-32.5), including 2 complete responses and 23 partial responses, in patients with advanced SCLC who had received a median of 2 prior lines of therapy. The median duration of response was 12.3 months. CRS was the most common adverse event, occurring in 52% of patients, though only one patient experienced grade 3 CRS (1%).^17^ These results were confirmed and expanded in the phase 2 DeLLphi-301 trial, where tarlatamab achieved a 40% ORR and a median overall survival of 14.3 months.^34^

Obrixtamig (BI 764532) is another half-life–extended IgG-like T-cell engager targeting DLL3. The first-in-human phase I trial (NCT04429087) evaluated its safety, tolerability, pharmacokinetics, and preliminary efficacy across DLL3-positive SCLC and neuroendocrine carcinomas. Early reports demonstrated manageable safety with CRS as the most frequent treatment-related adverse event (65%), generally low-grade and reversible, without reaching a maximum tolerated dose.^35^ Building on these findings, the phase II DAREON-5 study (NCT05882058) is currently assessing obrixtamig in patients with extrapulmonary neuroendocrine carcinomas (epNEC) and SCLC, with initial results suggesting encouraging antitumor activity (ORR 40% in patients with DLL3 high epNEC), including durable responses in heavily pretreated populations.

Another emerging DLL3-targeted engager is MK-6070 (also known as gocatamig or previously HPN328), a trispecific T-cell engager that binds DLL3, CD3, and albumin to extend half-life and improve tumor penetration. MK-6070 is being evaluated in an ongoing phase 1/2 clinical trial (NCT04471727) in patients with advanced cancers associated with DLL3 expression including SCLC, demonstrating preliminary evidence of manageable safety (characterized by CRS in 55% of patients [predominantly grade 1-2; and 1 grade 4], and immune effector cell-associated neurotoxicity syndrome in 7% [all grade 1-2]) and early signs of clinical activity across dose cohorts (showed in the 12 mg cohort an ORR of 44% [95% CI, 32-56] and a DCR of 66% [95% CI, 54-76] and an ORR of 55% [95% CI, 36-72] and a DCR 79% [95% CI, 61-91] in the 24 mg cohort) without reaching a maximum tolerated dose to date. Additionally, combination strategies exploring MK-6070 with DLL3-directed ADCs such as ifinatamab deruxtecan (ADC-B7H3) are under investigation in relapsed/refractory extensive-stage SCLC cohorts (NCT07227597).^36^

### Gp100 in uveal melanoma

Tebentafusp is an ImmTAC molecule that combines a high-affinity T cell receptor specific for gp100 presented by HLA-A*02:01 with an anti-CD3 effector domain. In a pivotal phase 3 trial in metastatic uveal melanoma (NCT03070392), tebentafusp significantly improved overall survival compared to investigator’s choice (median OS: 21.7 vs 16.0 months; HR 0.51), despite a low RECIST-defined ORR of ∼5%, highlighting a mechanism of action that extends beyond conventional tumor shrinkage and likely involves immune priming and modulation of the tumor microenvironment.^6^ This agent represented the first approved TCE for a solid tumor.

### STEAP1, KLK2 and PSMA in prostate cancer

Xaluritamig (AMG 509) is a half-life extended BiTE targeting STEAP1, a 6-transmembrane epithelial antigen overexpressed in prostate cancer. It binds STEAP1 on tumor cells and CD3 on T cells to induce cytotoxicity. In the ongoing phase 1 clinical trial (NCT04221542), preliminary results presented showed encouraging activity in patients with metastatic castration-resistant prostate cancer (mCRPC), including PSA decreasing in 49% of patients and confirmed radiographic responses in 41% of patients at higher dose levels. The safety profile was consistent with other TCEs, CRS being the most frequent adverse event.^37^

In addition to STEAP1, kallikrein-related peptidase 2 (KLK2) has emerged as an alternative prostate-restricted target for T-cell redirection. Pasritamig, a KLK2×CD3 bispecific antibody, is currently being evaluated both as a single agent and in combination with a PSMA×CD28 bispecific antibody designed to provide a costimulatory signal (signal 2). This combinatorial strategy aims to enhance T-cell activation and persistence while maintaining tumor specificity, addressing potential limitations of CD3-only engagement in the immunosuppressive prostate tumor microenvironment. In the first-in-human phase I study, pasritamig in monotherapy demonstrated proof-of-concept antitumor activity, with dose-dependent PSA declines and PSA reductions ≥50% observed in approximately 30% of treated patients at biologically active dose levels, in the absence of formally confirmed RECIST radiographic responses to date. The safety profile was characterized by manageable CRS, predominantly grade 1-2, supporting further clinical development and the exploration of coordinated signal 1 and signal 2 delivery strategies in prostate cancer.^38^

Finally, the prostate-specific membrane antigen (PSMA) has emerged as a major focus for T-cell redirection strategies in mCRPC given its high and relatively selective expression on prostate tumor cells. A half-life extended PSMA×CD3 bispecific T-cell engager, acapatamab (formerly AMG 160, NCT03792841**)**, has been evaluated in a first-in-human phase 1 study in patients with mCRPC refractory to androgen-receptor pathway inhibitors and taxane chemotherapy. Acapatamab demonstrated manageable safety with expected CRS and preliminary antitumor activity, including a confirmed PSA response (≥50% decline) in ∼30.4% of patients and radiographic partial responses in 7.4%, with a median PSA progression-free survival of approximately 3.3 months and median radiographic progression-free survival of 3.7 months in the dose expansion cohort, establishing proof-of-concept for PSMA×CD3 T-cell engagement in this disease.^39^

### MUC16 in ovarian and other MUC-16 positive tumors

Ubamatamab (REGN4018) is a MUC16×CD3 bispecific T-cell engager evaluated in recurrent epithelial ovarian cancer a disease characterized by high and homogeneous expression of MUC16 (CA-125). In a phase 1, first-in-human study (NCT03564340), ubamatamab with step-up dosing, showed an acceptable safety profile with CRS as the most frequent treatment-related adverse event, occurring in approximately 60-70% of patients, predominantly grade 1-2, and with grade ≥3 CRS reported in <5% of cases. Other treatment-related toxicities included fatigue, pyrexia, and gastrointestinal symptoms. Regarding efficacy, ubamatamab monotherapy showed limited but measurable antitumor activity, with confirmed ORR observed in ∼5-10% of heavily pretreated patients, and DCR of approximately 30-40%. Notably, enhanced activity was observed when ubamatamab was combined with the anti-PD-1 antibody cemiplimab, supporting the biological rationale for checkpoint inhibition to overcome T-cell exhaustion and immunosuppression in the ovarian tumor microenvironment. These data support a further development in MUC16-expressing tumors.^40^

### PRAME in HLA-A*02:01 restricted solid tumors

IMC-F106C is an ImmTAC that pairs a high-affinity TCR specific for PRAME/HLA-A*02:01 with an anti-CD3 effector domain. In an ongoing phase 1/2 basket trial (NCT05136706), early readouts showed a tolerable safety profile with CRS and skin-related toxicities (rash, pruritus) being the most frequent; CRS was predominantly grade 1-2, and grade ≥3 events occurred in a minority of patients and initial signals of antitumor activity across multiple PRAME-positive solid tumors confirmed by approximately 20-25% of evaluable patients, with DCR approaching 50%, despite a heavily pretreated population. These findings provide clinical proof of concept for TCR-based, HLA-restricted T-cell redirection targeting intracellular tumor antigens in solid tumors.^41^

### CLDN18.2 in advanced pancreatic ductal adenocarcinoma

IBI389 is a bispecific antibody targeting CLDN18.2 on tumor cells and CD3 on T cells. In a Phase I dose-escalation and expansion study (NCT05164458), it was evaluated in patients with advanced solid tumors, including pancreatic ductal adenocarcinoma (PDAC), with CLDN18.2 expression (≥10% of tumor cells with IHC 2+/3+). The safety profile was predictable, with most adverse events being grade 1-2; no grade ≥3 CRS was observed. Treatment-related grade ≥3 events in PDAC included elevated gamma-glutamyl transferase (20.3%), lymphocyte count decrease (9.4%), and nausea (7.8%). Preliminary efficacy signals were encouraging, with an ORR of approximately 30-31% and a DCR of around 70% among patients with significant CLDN18.2 expression. These findings support further investigation of IBI389 in CLDN18.2-positive gastrointestinal malignancies, including PDAC.^42^

In this context, ASP2138, a CLDN18.2×CD3 bispecific T-cell engager recently disclosed at ESMO, represents an additional emerging approach targeting CLDN18.2-positive gastrointestinal malignancies. In the phase I/Ib program, ASP2138 monotherapy showed an ORR of approximately 10%, with DCR observed in around 40% of treated patients. CRS occurred in approximately 30% of patients and was predominantly grade 1-2, with on-target gastrointestinal toxicity consistent with CLDN18.2 expression. Preliminary data from combination cohorts have also been reported, including ORRs of around 38% with paclitaxel and ramucirumab in the second-line setting and approximately 68% with FOLFOX plus pembrolizumab in the first-line setting, although these results remain early and exploratory. These findings further support CLDN18.2 as a clinically actionable target for T-cell redirection strategies in solid tumors.^43^

### Other solid tumor programs and emerging TCEs

A growing pipeline of other solid tumor–targeted TCEs is currently under investigation in early-phase clinical trials. These efforts focus on refining antigen selectivity, extending half-life, and incorporating conditionally activated designs to reduce off-tumor effects. Notable programs include:

- **CEAxCD3 bispecifics** (eg, cibisatamab, RO6958688 [NCT04826003]): developed for colorectal and gastrointestinal tumors. In a phase I trial, cibisatamab demonstrated an ORR of 20% in patients with metastatic CEA-positive colorectal cancer, with higher responses observed in tumors with high CEA expression.^44^ However, the development was discontinued due to significant gastrointestinal toxicity and limited clinical benefit.
- **HER2xCD3 bispecifics**: multiple bispecific formats targeting HER2 (eg, Runimotamab [RG6194], KN026, and novel HER2×CD3 TDBs) are in early-phase clinical development in HER2-positive solid tumors. In a phase I dose-escalation study of Runimotamab (alone or combined with trastuzumab) in metastatic HER2-positive breast cancer (NCT03448042), the combination arm showed encouraging antitumor activity and improved tolerability compared to monotherapy, with manageable infusion-related reactions and gastrointestinal toxicity.^45^ Preclinical studies of a HER2xCD3 asymmetric bispecific (M802) demonstrated potent cytotoxicity against HER2-high and -low tumors, including trastuzumab-resistant models, with a favorable therapeutic index in xenograft systems.^46^
- **MesothelinxCD3 TCEs**: AMG 427 (NCT03541369) showed dose-dependent activity but was ultimately discontinued due to mesothelin×CD3 T-cell engagers represent a promising therapeutic strategy in solid tumors with high mesothelin expression, including mesothelioma, pancreatic, and ovarian cancers. Among them, HPN536, a TriTAC-based construct with extended half-life, has demonstrated potent T cell–redirecting activity and favorable pharmacokinetic properties in preclinical studies, supporting its clinical development. This agent is currently under clinical evaluation (NCT03872206), although no mature clinical efficacy results have been published to date.^47^

Several next-generation TCEs have progressed into active clinical development, reflecting diversification of targets and platforms. HPN424 (PSMA×CD3), a TriTAC-XR technology, is under investigation (NCT03577028), with preliminary data demonstrating T-cell activation and tumor-specific pharmacodynamic effects.^48^ CTIM-76, a Claudin-6×CD3 bispecific antibody, is in development for germ cell and gynecologic malignancies, leveraging the tumor-restricted expression of Claudin-6 to enhance specificity.^49^ IBI343, a CLDN18.2×CD3 bispecific antibody developed by Innovent Biologics, is being evaluated in a phase I trial for patients with CLDN18.2-expressing tumors^50^ Other agents include MGD009 and MGD007, which target B7-H3 and gpA33 respectively, using the DART platform. MGD009 has completed a phase I trial (NCT02628535) showing acceptable safety but modest efficacy, while MGD007 was evaluated in NCT02248805 for gastrointestinal tumors.^51^^,^^52^ Finally, a portfolio of investigational agents, including VE-psitamab (AMG 199: CD3xMUC17), AMG 936 (CD3xILT3), AMG 305 (CD3×EGFR) and R5668 (MUC16xCD28), remains in early-stage development. These TCEs aim to overcome existing limitations by integrating conditionally active designs, optimized half-lives, and improved tumor selectivity.^9^  Table 1 summarizes the current clinical trials landscape.

**Table 1:** Clinical trials for T-cell engagers.

NCT code	TCE	Results	Current situation
**NCT01466179**	Blinatumomab (CD3xCD19)	Phase II study in relapsed/refractory B-cell ALL showing complete remission or CRh in ∼43% of patients, with frequent minimal residual disease clearance and manageable immune-related toxicity, supporting accelerated FDA approval.	Completed
**NCT03319940**	Tarlatamab (DLL3xCD3)	ORR 23.4% (95% CI, 15.7-32.5), incl. 2 CR and 23 PR; median DOR 12.3 mo; CRS in 52% (grade 3 in 1%)	Completed phase 1; efficacy signal confirmed in phase 2 (DeLLphi-301); under active clinical development in multiple SCLC settings
**NCT05361395**	Tarlatamab (DLL3×CD3) + PD-L1 inhibitor (durvalumab or atezolizumab)	In the DeLLphi-303 trial, maintenance therapy in SCLC showed DCR 62.5%, >85% 9-month survival, no unexpected toxicities	Ongoing phase 1/2
**NCT04429087**	Obrixtamig (BI 764532)—DLL3 × CD3 IgG-like T-cell engager	Full tumor regression in preclinical mouse models; phase 1 ongoing	Recruiting
**NCT05882058**	Obrixtamig (BI 764532)—DLL3 × CD3 TCE	In heavily pre-treated extrapulmonary neuroendocrine carcinoma (epNEC) patients with high DLL3 expression: ORR ∼40%, median duration of response ∼7.9 months	Phase 2 ongoing
**NCT03070392**	Tebentafusp (gp100 ImmTAC, HLA-A*02:01)	Phase 3 (IMCgp100-202): median OS 21.7 vs 16.0 mo; HR 0.51; low RECIST ORR (∼5%) with OS benefit.	Active, not recruiting; standard of care in mUM
**NCT04221542**	Xaluritamig (STEAP1×CD3, 2 + 1)	Early phase: PSA50 responses and RECIST PRs reported; manageable CRS per dose-esc.	Phase 3 recruiting
**NCT03564340**	Ubamatamab (MUC16×CD3) ± Cemiplimab	Phase 1: manageable safety (mostly low-grade CRS) and preliminary activity in recurrent EOC (conference reports).	Phase 1 ongoing
**NCT05136706**	IMC-F106C (PRAME ImmTAC, HLA-A*02:01)	Early signals of activity; tolerable safety	Recruiting/active
**NCT03448042**	Runimotamab (HER2×CD3) ± Trastuzumab	Phase I study showing limited activity with runimotamab monotherapy, while combination with trastuzumab achieved objective responses (∼20-30%) with a manageable safety profile and reduced CRS.	Phase 1 active, not recruiting
**NCT03541369**	AMG 427 (MSLN×CD3, HLE-BiTE)	No peer-reviewed efficacy posted; program prematurely discontinued per registry.	Discontinued
**NCT03872206**	HPN536 (TriTAC, MSLN×CD3)	Disease stabilization in subsets; safety acceptable; no results posted on registry.	Phase 1/2ª ongoing
**NCT04826003**	RO7122290 (FAP targeted 4-1BB) + Cibisatamab (CEACAM5xCD3)	Phase I study showed limited clinical activity and was discontinued due to dose-limiting gastrointestinal toxicity (severe enterocolitis/colitis), preventing further dose escalation and development	Completed
**NCT05740566**	Tarlatamab (DLL3×CD3) ± chemotherapy (carboplatine-etoposide)	In phase 3 SCLC, ORR 40%, median OS 14.3 months in phase 2; phase 3 results pending	Active, not recruiting
**NCT05090566**	Elranatamab (BCMA×CD3) + Standard myeloma agents (lenalidomide, daratumumab)	Preliminary phase 1/2 data show deep and durable responses in relapsed/refractory multiple myeloma	Ongoing
**NCT04586426**	Talquetamab (GPRC5D×CD3) + Teclistamab (BCMA×CD3)	Early phase 1 data: high ORR in heavily pretreated MM; CRS most common AE, manageable	Ongoing
**NCT04128423**	AMV564 (CD33xCD3) + anti–PD-L1 (Pembrolizumab)	Phase 1/2 study in AML/MDS showed acceptable safety; modest responses; combination arm recruiting	Active
**NCT05199372**	Tarlatamab (DLL3×CD3) + TKI (Cabozantinib)	No results posted	Recruitment completed
**NCT05164458**	IBI389 (CLDN18.2xCD3) ± inhibidor de PD-1 (IBI389: Sintilimab)	Phase 1: manageable safety with mainly grade 1-2 CRS and preliminary antitumor activity, with ∼30% ORR and ∼70% DCR in CLDN18.2-positive gastric/GEJ and pancreatic cancers.	Recruiting
**NCT03792841**	AMG160 (PSMAxCD3)	Early phase 1 study of a PSMA half-life extended bispecific (PSMA×CD3) showed safety and tolerability; no published efficacy results available publicly.	Terminated
**NCT04471727**	Gocatamig: HPN328/MK-6070 (DLL3xCD3) ± Atezolizumab	Interim phase 1/2 data for HPN328 (DLL3×CD3 TCE) showed manageable safety and preliminary antitumor activity, with around 40 % of evaluable patients having tumor shrinkage in small cell lung cancer or other DLL3-expressing neuroendocrine cancers.	Recruiting
**NCT03577028**	HPN424 (PSMA×CD3)	No results posted	Terminated
**NCT02628535**	MGD009 (B7-H3 (CD276)×CD3)	No results posted	Terminated
**NCT02248805**	MGD007 (gpA33 x CD3)	No results posted	Completed
**NCT06536049**	Epcoritamab (CD3xCD20) + Ibrutinib (BTKi)	No results posted	Recruiting
**NCT07082868**	Epcoritamab (CD3xCD20) + Ibrutinib (BTKi)	No results posted	Recruiting

## Biomarkers of response and resistance

Despite remarkable advances in immunotherapy, predictive biomarkers of response to TCEs remain limited, particularly in solid tumors. Current clinical tools such as PD-L1 expression, deficient mismatch repair (dMMR), and tumor mutational burden (TMB) correlate with benefit from ICIs in selected settings, but their predictive value for TCEs remains uncertain. Given the complex interplay between tumor biology and immune activation, combinatorial biomarker strategies integrating tumor and immune-related features may offer greater predictive power than single analytes.^53^^,^^54^

### Tumor antigen expression and heterogeneity

By design, TCEs depend on the presence of a TAA that can be targeted without inducing significant off-tumor toxicity. Therefore, antigen density, homogeneity, and tumor-specific expression are critical determinants of TCE efficacy.^9^ Tumor antigen heterogeneity or antigen loss, either at baseline or acquired, may result in resistance or relapse. Additionally, low-level expression in normal tissues can lead to on-target/off-tumor toxicity, especially in vital organs.

While antigen loss has been proposed as a mechanism of resistance, its role may vary by target. For example, longitudinal analyses of melanoma samples treated with tebentafusp did not demonstrate loss of gp100 expression, suggesting that other mechanisms, such as MHC-I downregulation, HLA blockade (tumor-intrinsic mechanisms that functionally impair HLA-mediated antigen presentation), or defective IFN-γ signaling, may underlie immune escape in some settings.^24^

### Tumor microenvironment and immune infiltration

Tumor-intrinsic resistance also arises from an immunosuppressive tumor microenvironment (TME). Features such as low T cell infiltration (“cold” tumors), high expression of regulatory T cells (Tregs), myeloid-derived suppressor cells (MDSCs), or checkpoint ligand upregulation (eg, PD-L1, PD-L2) can blunt T cell activation.^55^^,^^56^ Conversely, “hot” tumors with pre-existing TILs and immune activation signatures may be more responsive to TCEs. Accordingly, clinical trials combining TCEs with anti-PD-1 antibodies are ongoing to counteract immune exhaustion and improve efficacy.^17^

### HLA-restricted strategies and TCR-based TCEs

In the context of HLA-restricted TCEs, such as ImmTACs, HLA expression itself emerges as a critical predictive and eligibility biomarker.^6^^,^^57^ Pharmacodynamic markers such as serum CXCL10 elevation and early treatment-related rash development have been positively associated with survival, and may serve as on-treatment biomarkers of response.^24^^,^^58^ Other HLA-restricted TCEs under development (eg, IMC-F106C) are likely to rely on similar HLA-dependent and pharmacodynamic biomarker strategies.^41^

### Liquid biopsy and circulating biomarkers

Liquid biopsy technologies, including circulating tumor DNA (ctDNA) and ultradeep TCR sequencing, are emerging tools for dynamic longitudinal monitoring of response to TCEs. These approaches allow for the detection of minimal residual disease (MRD), clonal evolution, and early resistance events, potentially informing real-time treatment adaptation and therapeutic decision-making.^59^

### Immunogenicity and anti-drug antibodies

Anti-drug antibodies (ADAs) may compromise TCE efficacy by accelerating clearance and reducing target engagement. ADA formation has been reported more frequently with subcutaneous administration than intravenous routes, although clinical impact remains variable. As such, ADA development is therefore closely monitored during phase 1 dose-escalation trials, particularly for novel bispecific formats and engineered constructs.^60^

### Toxicity as biomarker

Toxicity, particularly the incidence and severity of CRS, emerge as a functional, pharmacodynamically informative, biomarker that provides insights into both target biology and therapeutic activity. For instance, TCEs directed against DLL3, have shown relatively low rates of high-grade CRS (only about 1-6% of patients developed grade 3 CRS in early trials) whereas TCEs targeting other antigens such as STEAP1 or PSMA appear to be associated with higher frequency and/or intensity of CRS events.^32^^,^^37^

These differences suggest that antigen density, tissue distribution, and binding kinetics may directly influence the observed toxicity profile. In addition, patient-related factors, including tumor burden and baseline immune status, may also modulate adverse event severity. Systematic integration of toxicity patterns into biomarker development frameworks could therefore help refine antigen selection, guide patient stratification, and improve the therapeutic window of TCEs in solid tumors.

## Toxicities, management, and monitoring strategies

TCEs are associated with a characteristic toxicity profile driven by rapid immune activation. Although these adverse events are generally manageable, they require careful anticipation, structured monitoring, and early intervention, particularly during the first treatment cycles. The spectrum and severity of toxicities vary depending on the target antigen, molecular format, dosing strategy, and patient-related factors such as tumor burden and prior therapies.^9^

The most frequent adverse event is CRS, which typically occurs within the first 72 hours to several days after treatment initiation or dose escalation. The incidence and severity generally decrease with subsequent administrations. Clinical manifestations range from low-grade fever and constitutional symptoms to hypotension, hypoxia, and organ dysfunction. In solid tumor trials, CRS is most commonly grade 1-2, with grade ≥3 events occurring less frequently than those reported with CAR T-cell therapies.^32^ Management follows established consensus guidelines and includes supportive care for mild cases, with early and prompt use of tocilizumab and corticosteroids for moderate to severe CRS.^61–63^ The implementation of consensus CRS grading criteria, step-up dosing schedules, prophylactic corticosteroids, and early use of IL-6 receptor blockade with tocilizumab has improved safety of TCEs^61^ and enabled safer dose escalation. These measures have allowed most contemporary investigational TCEs to be administered safely in phase I units with appropriate experience in immune effector cell therapies. The approval of tebentafusp further contributed to broader awareness of TCE-associated toxicities; however, given the rarity of metastatic uveal melanoma, real-world exposure to this agent remains limited in many oncology settings.^6^

Step-up dosing has emerged as a central mitigation strategy and is now incorporated into most of the late-stage TCE development programs. Nevertheless, specific step-up schedules, steroid prophylaxis, and routes of administration vary across compounds.^13^ Importantly, the incidence and severity of CRS vary considerably between TCE constructs, with no single determinant clearly accounting for these differences. Factors such as antibody format (eg, 1 + 1 versus 2 + 1), presence or absence of Fc-mediated half-life extension, binding affinity, target antigen density, tissue distribution, and tumor burden are all likely contributors.^13^^,^^32^^,^^61^^,^^62^ On the other hand, prophylactic blockade of the IL-6 pathway has emerged as an effective strategy to mitigate CRS. Experience derived from both hematologic and solid tumor TCE programs indicates that premedication with IL-6 receptor antagonists, most commonly tocilizumab and more recently sarilumab, can reduce the incidence and severity of CRS without compromising antitumor efficacy.^1–3^ In multiple early-phase studies, prophylactic or early pre-emptive administration of tocilizumab was associated with lower rates of grade ≥2 CRS, reduced corticosteroid requirements, and improved feasibility of dose escalation and outpatient administration.^64^ Sarilumab, which offers subcutaneous administration and a longer half-life, has demonstrated comparable pharmacodynamic control of IL-6–mediated inflammation and is increasingly being explored as an alternative strategy for CRS prevention, although prospective data in TCE-treated populations remain limited.^3^^,^^5^ Importantly, available evidence suggests that IL-6 blockade does not impair T-cell activation, target engagement, or depth of response, supporting its incorporation into standardized CRS prevention and management algorithms for selected T-cell engager platforms.^64–66^

Immune effector cell–associated neurotoxicity syndrome (ICANS) is less common with TCE than with CAR T-cell therapies but has been reported, particularly at higher dose levels or during early treatment cycles. Symptoms include headache, confusion, tremor, and, rarely, seizures. Management relies on close neurological monitoring, prompt corticosteroid administration in symptomatic patients, and temporary treatment interruption when clinically indicated.^62^

Hematologic toxicities, including lymphopenia, neutropenia, anemia, and thrombocytopenia, are frequently observed and may reflect both immune-mediated effects and cumulative bone marrow suppression from prior therapies. These cytopenias contribute to an increased infection risk, particularly in heavily pretreated patients. Supportive measures such as growth factor support and antimicrobial prophylaxis may be considered based on the severity of cytopenias and institutional practice, with a stronger evidence base and more standardized recommendations currently available in hematologic settings (eg, multiple myeloma treated with bispecific antibodies) than in most solid-tumor TCE programs.^13^

Monitoring strategies for T-cell engagers vary widely across institutions. Initial doses are frequently administered in an inpatient setting, particularly for first-in-human studies, high-risk targets, or patients with high tumor burden. With increasing clinical experience, improved mitigation strategies, and greater familiarity with toxicity patterns, outpatient administration with close monitoring has become feasible for selected patients and later treatment cycles.^61^^,^^62^ Standard monitoring protocols typically include frequent vital sign assessments, serial laboratory evaluation of inflammatory markers, and early recognition of neurological symptoms. Harmonization of monitoring strategies and development of risk-adapted algorithms will be critical as T-cell engagers transition toward broader clinical implementation in solid tumors.

## Future perspectives

The rapid development of TCEs has opened new therapeutic opportunities in oncology, especially in solid tumors where conventional immunotherapy often fails. Solid tumors present a unique set of challenges for TCE therapy that limit efficacy and complicate clinical translation. Tumor-intrinsic features such as antigen heterogeneity, low immunogenicity, hypoxia, and a dense stromal architecture or an acidic microenvironment pose significant barriers to effective T cell infiltration and cytotoxic engagement.^55^^,^^56^ Furthermore, immune evasion mechanisms characterized by marked sparse tumor-infiltrating lymphocytes (TILs), T cell exhaustion, and the upregulation of checkpoint molecules like PD-1, CTLA-4, and LAG-3 can suppress T cell function, leading to reduced cytokine secretion and impaired cytotoxicity.^67–70^ In parallel, safety concerns such as CRS and neurotoxicity continue to represent key limiting factors, necessitating vigilant monitoring and continued refinement of dosing and mitigation strategies.^32^^,^^62^^,^^66^

These biological and clinical hurdles have spurred a wave of innovation aimed at enhancing the precision, efficacy, and tolerability of TCEs in solid tumors. Emerging platforms integrate conditionally activated designs, multispecific targeting strategies, and half-life extension technologies. When coupled with rational combinatorial regimens and biomarker-driven patient stratification, these strategies are poised to define the next frontier of T cell–redirecting therapies in oncology.

### Novel engineering strategies for safer and more effective TCEs

The development of TCEs in solid tumors faces 2 major biological hurdles: the risk of on-target/off-tumor toxicity due to shared antigen expression in normal tissues, and the immunosuppressive tumor microenvironment. To address these challenges, several innovative engineering approaches are being explored.

Conditional activation strategies, such as Probody TCEs, represent a key advance. These molecules remain systemically inactive through peptide-based masking of the CD3 or TAA binding domains, which is released only upon cleavage by tumor-associated proteases such as matrix metalloproteinases (MMPs) or cathepsins. This tumor-restricted activation reduces systemic toxicity, limits CRS, and improves the therapeutic window by restricting CD3 engagement to the tumor microenvironment.^71^^,^^72^

CX-904, a protease-activated, masked EGFR×CD3 Probody TCE, was designed to remain systemically inactive until antibody binding is unmasked by tumor microenvironment–associated proteases. This strategy aimed to restrict CD3 engagement to tumor sites and reduce off-tumor toxicity. Although early phase 1 data suggested a favorable safety profile, with no grade ≥2 CRS events and preliminary signs of activity in pancreatic cancer patients, the clinical development of CX-904 has since been discontinued.^71^ Similarly, XPAT proteins targeting HER2 and EGFR have shown promising preclinical safety, substantially improved safety margins compared to active TCEs in non-human primates.^73^

Other strategies, including TRACTr, ProTriTAC, and Protect, use either small peptides, albumin-binding domains, or inhibitory receptor ligands (eg, PD-1/PD-L1) to achieve tumor-selective CD3 engagement, extend systemic exposure, and reduce systemic exposure.^71^^,^^74^^,^^75^ Additional mechanisms under investigation include tumor-specific physicochemical triggers, such as acidic pH and ATP concentration as activators of masked TCEs.^76^

### Expanding functional payloads: arming the engagers

Next-generation TCEs aim not only to redirect T cells, but also to modulate their function through cytokine delivery or co-stimulation. For example, the GPS (guided pMHC staging) system fuses an IL-2 mutein to a pMHC-specific TCE to selectively stimulate memory CD8^+^ T cells, with reduced CRS in preclinical models.^77^ Similarly, TriKEs integrate a CD16- or CD3-binding domain with a stimulatory cytokine such as IL-15 to promote NK/T cell expansion, survival and effector function.^77^

Clinical trials are already exploring these modalities: the EGL-001 study evaluates an anti-CTLA-4 antibody fused to a Treg-selective IL-2R antagonist IL-2 mutein. Although still in early phases, it represents a proof-of-concept for functionalized armed immunotherapies in immunosuppressive tumor contexts (NCT066822486).

### Synergistic combinations

Combination strategies are being investigated to overcome the immunosuppressive microenvironment and improve TCE efficacy:

- Checkpoint inhibitors such as anti–PD-1/PD-L1 and anti–CTLA-4 can reinvigorate exhausted T cells and work synergistically with TCEs. Trials like DeLLphi-303 (NCT05361395), combining tarlatamab with durvalumab or atezolizumab in SCLC maintenance, show a DCR of 62.5%, with survival >85% at 9 months and no unexpected toxicities.
- Chemotherapy can enhance tumor antigenicity and reduce immunosuppressive cells like Tregs, potentially boosting TCE activity. Early-phase trials, such as NCT05740566 (tarlatamab + carboplatin-etoposide in SCLC) and NCT05090566 (REGN5837 + chemotherapy in myeloma), are exploring these combinations. However, agents like capecitabine may upregulate PD-1 expression, requiring careful sequencing.^78^ Importantly, clinical experience with blinatumomab in B-cell acute lymphoblastic leukemia provides a strong rationale for a sequential chemotherapy–TCE strategy. In this setting, blinatumomab has shown optimal efficacy when administered as maintenance therapy following induction chemotherapy, where cytoreduction leads to a lower tumor burden and a consequent reduction in the incidence and severity of cytokine release syndrome, thereby improving safety. Moreover, because blinatumomab is capable of eliminating non-cycling malignant cells, it has demonstrated a unique ability to induce minimal residual disease (MRD) negativity in patients who remain MRD-positive after chemotherapy. Applying a similar post-chemotherapy maintenance approach with TCEs may be particularly relevant in small cell lung cancer, where residual disease after induction therapy could represent an optimal setting for immune-mediated tumor clearance.^79^^,^^80^
- Co-stimulatory agonists, targeting CD28, 4-1BB, or OX40 aim to enhance T cell activation and persistence when used in combination with CD3-directed TCEs. Engagement of CD3 alone (signal 1) may lead to suboptimal T-cell responses, functional exhaustion, or activation-induced cell death, particularly within the immunosuppressive tumor microenvironment of solid tumors. Providing a controlled costimulatory signal (signal 2) has therefore emerged as a rational strategy to improve the depth and durability of antitumor responses. Preclinical studies have consistently shown that combining TCEs with CD28 or 4-1BB agonism enhances T-cell proliferation, cytokine production, cytotoxic activity, and antitumor efficacy compared with CD3 engagement alone. Importantly, tumor-restricted or conditional delivery of costimulatory signals appears critical to mitigate the risk of systemic T-cell activation and severe toxicity. Preclinical data support this synergy, and early clinical trials such as NCT04586426 (Talquetamab + Teclistamab) and NCT04128423 (CD33xCD3 + Pembrolizumab) are assessing feasibility and safety.^27^
- Combination with tyrosine kinase inhibitors (TKIs) has been primarily tested in hematological neoplasms with BTK inhibitors like ibrutinib + epcoritamab (NCT06536049, NCT07082868), glofitamab + ibrutinib ± obinutuzumab (NCT06357676) and glofitamab + acalabrutinib (NCT06054776). In solid tumors, NCT05199372 is combining Tarlatamab with cabozantinib, aiming to modulate the tumor microenvironment and enhance TCE activity.
- Oncolytic viruses (OVs) promote local T cell responses by releasing tumor antigens, enhancing TCE function. Preclinical OV-BiTE trials suggest synergy with CPIs and reduced systemic toxicity due to tumor-specific replication.^81^

## Discussion

TCEs represent a transformative class of immunotherapeutic agents capable of bypassing classical antigen presentation mechanisms and directly redirecting immune effector cells toward tumor cells. Their clinical success in hematologic malignancies, particularly with CD19-directed therapies such as blinatumomab, has not only established proof of concept but also demonstrated that TCE-mediated redirection of T cells can achieve meaningful improvements in disease eradication and even cure rates in acute lymphoblastic leukemia. Translating comparable efficacy into solid tumors has proven more complex.

The challenges posed by solid tumors (including heterogeneous antigen expression, physical barriers within the TME, and a high propensity for immune evasion) have necessitated more sophisticated TCE platforms. Novel engineering strategies such as conditionally activated constructs (eg, Probody, XPAT, TRACTr, Protect) may offer enhanced tumor selectivity, improved pharmacokinetics, and a more favorable safety profile, mitigating the risk of systemic toxicity such as CRS, although clinical evidence of this is still missing. In addition, armed TCEs that incorporate cytokine signaling or co-stimulatory domains promise to not only enhance cytotoxicity but also reshape the immune contexture within the TME.

Despite these advances, clinical data in solid tumors remain largely limited to early-phase trials. Among the most encouraging results to date, DLL3-targeted agents like tarlatamab have demonstrated durable responses and manageable toxicity in SCLC, setting a precedent for the broader application of TCEs in other solid tumors. Promising results are being seen in tumor types such as prostate, ovarian, and gastrointestinal cancers, though late phase studies are still missing.

One of the key determinants of TCE success in solid tumors will be biomarker-guided patient selection. The identification of predictive biomarkers, ranging from tumor-associated antigen expression to tumor-infiltrating lymphocytes, protease activity, HLA status, and serum chemokines, will be essential to optimize benefit. Moreover, adaptive resistance mechanisms such as antigen loss, immune checkpoint upregulation, or IFN-γ signaling disruption underscore the need for rational combination strategies, particularly with checkpoint inhibitors and chemotherapy.

In this context, it is also relevant to position T-cell engagers relative to other emerging anticancer modalities. T-cell engagers should be considered alongside other rapidly expanding anticancer modalities, particularly antibody–drug conjugates (ADCs) and cell-based therapies. Compared with ADCs, T-cell engagers offer a distinct mechanism of action that does not rely on cell cycle progression. By redirecting cytotoxic T cells, TCEs can in principle eliminate non-cycling or dormant tumor cell populations, which may be less susceptible to payload-based ADCs such as topoisomerase I inhibitors that preferentially target proliferating cells. We also envision potential future combinations of TCEs and ADCs.

When compared with cell-based therapies such as CAR T cells or engineered TCR-T cells, TCEs offer important practical advantages, including off-the-shelf availability, simplified manufacturing, and more flexible dosing and treatment discontinuation. However, this ease of use comes at the cost of chronic administration, reduced cellular persistence, and, in some cases, more limited durability of response compared with adoptively transferred cellular products. Cell therapies may achieve long-term disease control through in vivo expansion and persistence, after one single dose, but are associated with complex logistics, higher upfront toxicity risk, and restricted accessibility outside specialized centers.

Emerging technologies aim to further expand the therapeutic scope of T-cell redirection while addressing some of these limitations. Among these, mRNA-based bispecific platforms represent a novel approach to transient, controllable T-cell engagement. BNT142, an mRNA-encoded CLDN6×CD3 bispecific antibody, exemplifies this strategy by enabling in vivo production of a short-lived T-cell engager, potentially improving safety through temporal control while retaining antitumor activity. Whether such platforms can achieve sufficient efficacy and durability in solid tumors remains to be determined, but they illustrate the continued diversification of T-cell–engaging technologies.

## Conclusion

TCEs are emerging as a transformative immunotherapeutic modality in solid tumors. While their clinical efficacy is well-established in hematologic malignancies, the application of TCEs to solid tumors remains in its early stages and presents unique biological and technical challenges. Recent advances in molecular engineering have enabled the development of next-generation TCEs aiming to improve tumor selectivity, pharmacokinetics, and safety. The success of agents like tarlatamab in small cell lung cancer has validated the potential of this approach and sparked the clinical investigation of multiple candidates across a variety of solid tumor types. However, durable efficacy and broader applicability will likely depend on the identification of predictive biomarkers, such as tumor-associated antigen expression, protease activity, HLA status, or immune infiltration profiles, as well as on the implementation of rational combination strategies with checkpoint inhibitors, chemotherapy, or oncolytic platforms. Overall, TCEs hold substantial promise to expand the reach of immunotherapy in solid tumors. As our understanding of resistance mechanisms deepens and clinical experience accumulates, these agents may become a fundamental pillar in the therapeutic landscape for selected patient populations.

## Data Availability

Not applicable.
